# Effect of mouth rinses on roughness and optical properties of restorative materials for oral rehabilitation

**DOI:** 10.2340/biid.v12.43204

**Published:** 2025-03-13

**Authors:** Laura F. Carvalho, Edmara T. P. Bergamo, Ernesto B. Benalcázar-Jalkh, Tiago M. B. Campos, Abbas Zahoui, Elisa de Souza Fermino, Ana Clara Mota de Oliveira, Ana Carolina Magalhães, Estevam A. Bonfante, Fábio José B. Bezerra, Larissa M. M. Alves

**Affiliations:** a Department of Prosthodontics and Periodontology, Bauru School of Dentistry, University of São Paulo, Bauru, Brazil; b Biomaterials Division, New York University College of Dentistry, New York, NY, USA; c Department of Biological Sciences, Bauru School of Dentistry, University of São Paulo (USP), Bauru, Brazil; d Department of Biological Sciences, São Paulo State University (UNESP), Botucatu, Brazil

**Keywords:** CAD-CAM, acrylic resin, mouth rinse, translucency, roughness

## Abstract

The objective of this study was to evaluate the influence of mouth rinses on the roughness and optical properties of three polymeric restorative materials. Cylinders were obtained from Ivotion Dent, Ivotion Base, and Empress Direct. Nano-hybrid composite teeth were also investigated (SR Phonares II). Specimens were divided into four subgroups for mouth rinsing simulation according to the mouth rinse: (1) Distilled water; (2) Soft-Tissue; (3) Implants/Cosmetic; and (4) PerioGard. Roughness (Ra) and optical properties were evaluated before and at timepoints during mouth rinsing simulation. Roughness increased after rinsing simulation for Ivotion Dent and Ivotion Base after all mouth rinses. Soft-Tissue and Cosmetic increased the Ra of Empress Direct. Translucency parameter (TP) of Ivotion Dent and Empress Direct increased, while Contrast ratio (CR) values decreased after rinsing simulation, regardless of the solution used. Ivotion Base demonstrated higher TP after rinsing with Soft-Tissue and Implants mouth rinses compared to the control group, while no difference among them was observed for CR. The mouth rinses affected the roughness and optical properties of materials differently after the rinsing simulation.

## Introduction

The CAD/CAM (Computer-Aided Design and Computer-Aided Manufacturing) technology has facilitated notable advancements in oral rehabilitation procedures. The milling of veneers, crowns, partial prostheses, abutments, and splints has been increasing since the 90s [1]. Recently, the Ivoclar Vivadent company introduced an innovative system for the fabrication of digital dentures. The Ivotion system combines in one disc both high-quality tooth and base materials for the design of removable or implant-supported complete dentures.

These technology advancements enable efficient dental replacement with esthetic significance [1, 2]. Nevertheless, dental materials are constantly exposed to pH oscillation, temperature changes, acid solution, and moisture, which can influence their properties [3]. Besides physiology and diet, oral care products also contain components, such as alcohol in different concentrations and antimicrobial agents, which may influence mechanical and optical properties of dental materials [4, 5]. Mouth rinses are frequently used for their antimicrobial and anti-inflammatory effects, which complement mechanical biofilm control [6]. Nevertheless, due to the ease of their acquisition, the use of these products is often not prescribed or followed by professional supervision. In implantology and periodontology, mouth rinses with chlorhexidine are widely prescribed in periodontal and post-surgical therapy [7], however, with an adverse effect of staining on teeth and prosthetic materials [8]. Previous studies have reported changes in optical properties and surface roughness of materials in contact with acidic solutions [9–11], as well as color alterations due to the use of oral hygiene mouth rinses [12–14].

Simultaneously with the constant development of new dental materials for a wide range of clinical scenarios, new products for dental care are also introduced to the market with different compositions and clinical indications. Therefore, it is essential to assess alterations in the optical and physical properties of polymeric materials used in implant-supported complete prostheses, as well as in denture teeth, as these alterations can make the surface of the prosthesis more prone to biofilm accumulation, potentially facilitating the onset of peri-implant disease and compromising the esthetics of the rehabilitation [15]. Additionally, considering the progressive use of digital workflows in full-mouth rehabilitations, it becomes essential to characterize the interaction of these factors and their influence on the surface features of acrylic resins provided as discs for milling in CAD/CAM systems. Moreover, in oral rehabilitation, the association of prosthetics and direct restorative treatments is frequently employed to reestablish functionality, health, and esthetics, which results in materials of different classes, compositions, and manufacturing techniques exposed to oral environment and products for daily oral hygiene, with a wide variety of chemical composition. Hence, it is pivotal to assess the alterations in materials resulting from the use of mouth rinses, ensuring that prescriptions align appropriately with the specific restorative conditions of each case. This highlights the need to investigate the effects of novel products on the properties of different resin-based dental materials.

This study aimed to evaluate the influence of mouth rinses with different compositions on the surface roughness and optical properties of different polymeric materials, including CAD/CAM acrylic resins, resin composite, and composite denture teeth. The postulated null hypothesis was that there would be no significant differences on the surface roughness and optical properties of the materials before and after mouth rinse exposure regardless of the composition of the mouth rinse.

## Materials and methods

Manufacturers and composition of all materials are shown in Table 1.

**Table 1:** Manufacturers, composition, and lot number of the materials.

Product name	Manufacturer	Composition	Lot number
Ivotion Base	Ivoclar, Schaan, Liechtenstein.	Polymethyl methacrylate (PMMA) > 90%; co-polymer, pigments	Z00J71
Ivotion Dent	Ivoclar, Schaan, Liechtenstein.	Double crosslinked polymethyl methacrylate (PMMA)	Z00Y24
Empress Direct	Ivoclar, Schaan, Liechtenstein.	Barium glass, mixed oxide, Ba-Al-fluorosilicate glass (78.1%); Dimethacrylate (21.5%); Catalysts and stabilizers (0.4%); Pigments (< 0.1%)	Z03SFN
SR Phonares II	Ivoclar, Schaan, Liechtenstein.	Nano-Hybrid Composite	-
Cosmetic Mouth rinse	N&W dental care, São Paulo, Brazil.	Aqua, Glycerin, Hydrogenated Castor Oil, Xylitol, Lauryl Glucoside, Soudium Lauryl Sulfate, Silanediol, Salicylayte, Flavor, PVP, Tetrasodium Pyrophosphate, Camellia Sinensis Leaf Extract, Sodium, Benzoate, Hyaluronic Acid, Disodium Pyrophosphate, Melaleuca Alternifólia Leaf Extract, Sodium Monofluorophosphate, Dissodium, EDTA, Sodium Saccharin, Sodium Fluoride (226ppm), Cl 16185, Cl42090.	-
Implants Mouth rinse	N&W dental care, São Paulo, Brazil.	Aqua, Glycerin, Hydrogenated Castor Oil, Lauryl Glucoside, Sodium Lauryl Sulfate, Flavor, PVP, Tetrasodium Pyrophosphate, Xylitol, Camellia Sinensis Leaf Extract, Sodium Bonzoate, Hyaluronic Acid, Disodium Pyrophosphate, Melaleuca Alternifólia Leaf Extract, Silanediol Salicylate, Disodium EDTA, Sodium Saccharin, Cl 16185, Cl42090.	DEM1754
Soft Tissue Mouth rinse	N&W dental care, São Paulo, Brazil.	Aqua, Glycerin, Hydrogenated Castor Oil, Hyaluronic Acid, Soudium Lauryl Sulfate, Camellia, Sinensis Leaf Extract, Lauryl Glucoside, Flavor, Tetrasodium Pyrophosphate, PVP, Xylitol, Sodium Benzoate, Melaleuca Alternifólia Leaf Extract, Silanediol Salicylayte, Disodium Pyrophosphate, Sodium Saccharin, Disodium, EDTA, Cl16185, Cl42090.	DEM1756
PerioGard	Colgate-Palmolive Company, New York, USA	0.12% gluconate (or digluconate formulation with chlorhexidine-free concentration (0.067%), water, glycerin, ethanol, polysorbate 20, aromatic composition with predominant flavor of mint, sodium saccharine, FD&C blue dye #1	

### Specimen preparation

Two PMMA (Polymethyl Methacrylate) CAD/CAM discs (Ivotion Dent and Ivotion Base) and one light-curing resin composite (Empress Direct), from the same manufacturer (Ivoclar Vivadent, Schaan, Liechtenstein), were tested in this study. Nano-hybrid composite teeth, SR Phonares II (Ivoclar Vivadent), were also investigated, in their entity shape (*n* = 48). To prepare the specimens, the CAD/CAM materials (Ivotion Dent and Ivotion Base) were milled from their respective pucks in cylinders (3 mm of diameter and 20 mm of height). Subsequently, the cylinders were cut in a precision water-cooled machine (IsoMet 1000; Buehler, Lake Bluff, IL, USA) to obtain cylinders with 3 mm of diameter and 3 mm of thickness (*n* = 48/material). Resin composite specimens (Empress Direct; *n* = 48) were prepared using a metallic matrix with 3 mm of thickness and 3 mm of diameter. A single increment of resin composite was inserted into the matrix using a spatula (Suprafill #1; Duflex, Juiz de Fora, Brazil) and light cured for 40 s using an LED (Light Emitting Diode) device (VALO Corded; Ultradent, Utah, USA; 1,000 mW/cm^2^), as per manufacturer’s instructions. All specimens were smoothed with #600, 1,200, 2,500, and 4,000-grit SiC paper [16].

### Simulated mouth rinsing

**Figure 1:**
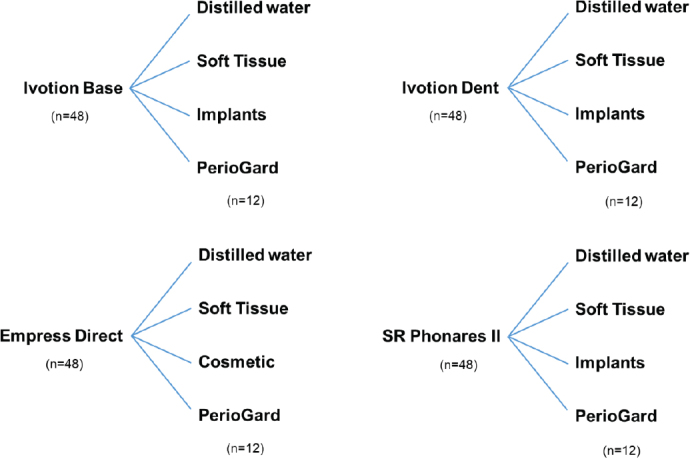
Design of the study and distribution of groups according to material and mouth rinse.

Four different mouth rinses were tested: Soft Tissue, Implants, Cosmetic, and PerioGard. According to the manufacturer, the three N&W mouth rinses have different characteristics and clinical indications, with the primary ones as follows: Soft tissue is indicated for pre- and post-operative oral surgery care and daily use to prevent and treat oral diseases. Implants are indicated for pre- and post-operative care and daily use to prevent oral diseases in patients with dental implants. Cosmetic is indicated for pre- and post-operative care and daily use to prevent oral diseases, with double fluoride. Distilled water was used as a control. The specimens of each restorative material were divided into different subgroups according to the mouth rinse solution and their clinical indications and rehabilitative treatments (*n* = 12) (Figure 1). Ivotion Base, Ivotion Dent, and SR Phonares II were tested considering scenarios involving implant-supported total prostheses and post-surgical care, using Soft Tissue and Implants. For Empress Direct, Cosmetic and Soft Tissue were selected to reflect its application in patients with resin composite restorations and post-surgical procedures. PerioGard is a commercially chlorhexidine-based rinse (containing 0.12% chlorhexidine or equivalent) that was included due to its wide recognition and use for general oral health care. The specimens were placed in plastic recipients containing 20 ml of the respective solution, submerged in a 37° water bath. Mouth rinsing was simulated using an orbital shaker table (Tecnal Equipamentos Cientificos, Piracicaba, Brazil). To simulate 2 min of mouth rinsing per day for 6 months, a total of 6 h of simulated rinsing was used [17, 18], considering the worst-case scenario of using non-surgical periodontal therapies with evaluation stages (T0, T1, T2, and T3) based on the time intervals used in these therapies, being T0 at baseline, T1 after 1:30 h, T2 after 3 h, and T3 after 6 h of rinsing [7]. After each cycle of rinsing, the specimens were washed abundantly with distilled water and dried and then subjected to testing.

### Surface roughness

Each specimen was scanned in five equidistant surface points (2.5 mm of reading, 250 μm apart) using a contact profilometer (Mahr Perthometer; Göttingen, Germany) to determine the roughness (Ra parameter) with 0.8 mm cut-off. The mean Ra for each specimen (*n* = 12) was calculated before (T0) and after mouth rinse simulation (T3).

### Optical properties

The contrast ratio (CR) and translucency parameter (TP) by color difference (ΔE) measurements were determined using parameters obtained by reflectance tests performed with a bench top spectrophotometer (CM 3700d Konica Minolta, Tokyo, Japan). Six specimens of each group were placed on black (b) and white (w) backgrounds cards for determining the reflectance values and CIE L*a*b* color coordinates with a wavelength of 400–700 nm. CR is the property that measures the transparency or opacity of the material by the ratio of reflectance of the specimen on the black background (Yb) to the reflectance of the same specimen on a white background (Yw), which is given by *CR* = *Yb/Yw.* TP, which defines the masking ability of the material, was obtained through the calculation of the color difference parameter CIEDE2000 (Δ*E*_00_) of the reflectance tests of the specimens over the black and white backgrounds, according to the formula:


△E00=[△L′KLSL]2+(△C′KCSC)2+(△H′KHSH)2+RT(△C′KCSC)(△H′KHSH)12,

where, L, C and H correspond to the difference in lightness, chromaticity, and hue for the specimens. R_T_ is a rotation function that accounts for the interaction between chroma and hue differences in the blue region. Weighting functions S_L_, S_C_, and S_H_ adjust the total color difference for variation in the location of the color difference pair in L’, a’, and b’ coordinates, and the parametric factors K_L_, K_C_ and *K_H_* are correction terms for deviations from reference experimental conditions [19–21]. The test was performed in the specimens before (T0) and after (T1, T2, and T3) mouth rinsing.

### Statistical analyses

Ra data were statistically evaluated using repeated measures analysis of variance followed by post-hoc comparisons by Tukey test, at a significance level of 5%. CR and TP data were statistically evaluated using analysis of variance followed by post-hoc comparisons by Tukey test, at a significance level of 5%. The statistical analyses were performed using GraphPad Prism software (GraphPad Software Inc., San Diego, USA).

## Results

### Roughness

**Figure 2:**
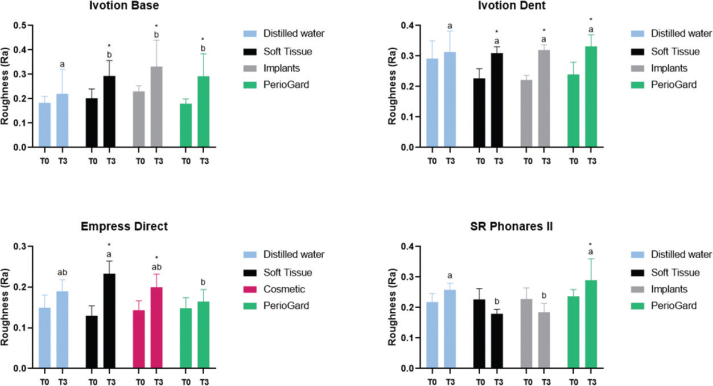
Mean and standard deviation for Ra surface roughness parameter of the materials as a function of mouth rinse and timepoints. T0: before mouth rinsing simulation; T3: after mouth rinsing simulation. Different letters indicate statistical difference between materials under the same timepoint (T3). *Statistical difference between the timepoints (T0*T3) for each mouth rinse.

Mean and standard deviation values of Ra as a function of the mouth rinse and timepoints are presented in Figure 2. The statistical analysis revealed a significant increase in surface roughness after all mouth rinsing simulation for Ivotion Dent and Ivotion Base over timepoint comparisons (*p* < 0.05), whereby no difference was observed among the mouth rinses. The Soft Tissue and Cosmetic increased the Ra of Empress Direct, whereas they promoted similar values to distilled water. The roughness of the SR Phonares II artificial teeth increased only after rinsing with PerioGard, while Soft Tissue and Implants promoted the lowest Ra values (*p* > 0.05).

### Optical properties

**Figure 3:**
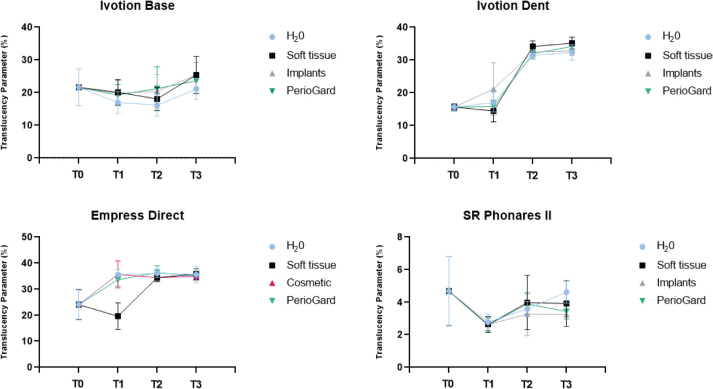
Mean and standard deviation for translucency parameter (TP) of the materials as a function of mouth rinse and timepoints.

**Figure 4:**
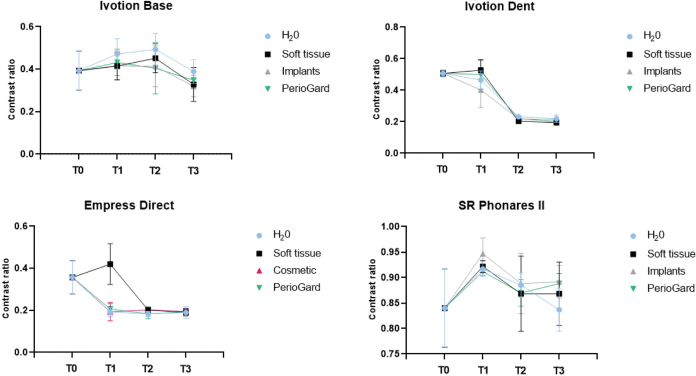
Mean and standard deviation for contrast ratio of the materials as a function of mouth rinse and timepoints.

Mean and standard deviation values of TP and CR as a function of the mouth rinse and timepoints are presented in Tables 2 and 3. The data are also represented in graphs in Figures 3 and 4. Statistical analysis demonstrated that TP values of Ivotion Dent and Empress Direct significantly increased by mouth rinsing simulation over timepoints regardless the mouth rinse (*p* < 0.05), with similar values among the groups for the respective materials (T3) (*p* > 0.05). Ivotion Base demonstrated significantly higher TP values after rinsing simulation (T3) with Soft Tissue and Implants compared to the control group (*p* < 0.05).

**Table 2:** Translucency parameter mean values and standard deviation (SD) for the materials according to the mouth rinse and timepoints and ANOVA and Tukey test.

Translucency parameter – Mean ± SD					
		T0	T1	T2	T3
Ivotion Base	Distilled water	21.6 ± 5.6 Aa	17 ± 3.3 Ab	16.1 ± 3.3 Ab	21.1 ± 3.2 Aa
	Soft Tissue	21.6 ± 5.6 Aab	20.1 ± 3.8 Aa	18 ± 3.5 ABa	25.4 ± 5.6 BCb
	Implants	21.6 ± 5.6 Aab	19.8 ± 3.8 Aa	20.4 ± 5.1 Ba	25.4 ± 3.8 BCb
	PerioGard	21.6 ± 5.6 Aab	19.2 ± 3.2 Aa	21.2 ± 6.6 Bab	23.6 ± 2.5 ACb
Ivotion Dent	Distilled water	15.6 ± 1.1 Aa	17 ± 2.9 Aa	31.4 ± 1.1 Ab	32.3 ± 2.4 Ab
	Soft Tissue	15.6 ± 1.1 Aa	14.4 ± 3.2 Aa	34.1 ± 1.7 Ab	35.1 ± 1.8 Ab
	Implants	15.6 ± 1.1 Aa	21.1 ± 7.9 Bb	32.4 ± Ab	32.8 ± 1.3 Ab
	PerioGard	15.6 ± 1.1 Aa	15.8 ± 4.7 Ab	32 ± 1 Ac	34.1 ± 1 Abc
Empress Direct	Distilled water	24.1 ±5.7 Aa	35.6 ± 1.9 Ab	36.1 ± 1.6 Ab	35.6 ± 3.1 Ab
	Soft Tissue	24.1 ± 5.7 Aa	19.6 ± 5.1 Bb	34.4 ± 1.5 Ac	35.8 ± 2 Ac
	Cosmetic	24.1 ± 5.7 Aa	35.7 ± 5.1 Ab	34.4 ± 1 Ab	35 ± 1.4 Ab
	PerioGard	24.1 ± 5.7 Aa	33.6 ± 2.4 Ab	36.3 ± 2.6 Ab	35.2 ± 1.7 Ab
SR Phonares II	Distilled water	4.6 ± 2.1 Aa	2.7 ± 0.5 Aa	3.5 ± 0.4 Aa	4.6 ± 0.6 Aa
	Soft Tissue	4.9 ± 2.1 Aa	2.6 ± 0.4 Aa	3.9 ± 1.6 Aa	3.9 ± 1.4 Aa
	Implants	4.9 ± 2.1 Aa	2.6 ± 0.3 Aa	3.2 ± 1.3 Aa	3.2 ± 0.7 Aa
	PerioGard	4.6 ± 2.1 Aa	2.5 ± 0.4 Aa	3.9 ± 0.6 Aa	3.4 ± 0.4 Aa

*Different uppercase letters indicate statistical difference between mouth rinse under the same timepoint for each material. Different lowercase letters indicate statistical difference between timepoints under the same mouth rinse for each material.

**Table 3:** Contrast ratio mean values and standard deviation (SD) for the materials according to the mouth rinse and timepoints and ANOVA and Tukey Test.

Contrast ratio – Mean ± SD					
		T0	T1	T2	T3
Ivotion Base	Distilled water	0.3 ± 0.09 Aa	0.4 ± 0.07 Ab	0.4 ± 0.07 Ab	0.3 ± 0.05 Aa
	Soft Tissue	0.3 ± 0.09 Aab	0.4 ± 0.06 Aa	0.4 ± 0.06 ABa	0.3 ± 0.07 ABb
	Implants	0.3 ± 0.09 Aa	0.4 ± 0.06 Aa	0.4 ± 0.09 Ba	0.3 ± 0.05 Bb
	PerioGard	0.3 ± 0.09 Aab	0.4 ± 0.06 Aa	0.4 ± 0.1 Bab	0.3 ± 0.04 ABb
Ivotion Dent	Distilled water	0.5 ± 0.02 Aa	0.4 ± 0.05 ABa	0.2 ± 0.01 Ab	0.2 ± 0.02 Ab
	Soft Tissue	0.5 ± 0.02 Aa	0.5 ± 0.06 Aa	0.2 ± 0.01 Ab	0.1 ± 0.01 Ab
	Implants	0.5 ± 0.02 Aa	0.4 ± 0.1 Bb	0.2 ± 0.02 Ac	0.2 ± 0.01 Ac
	PerioGard	0.5 ± 0.02 Aa	0.5 ± 0.09 Aa	0.2 ± 0.01 Ab	0.2 ± 0.01 Ab
Empress Direct	Distilled water	0.3 ± 0.07 Aa	0.1 ± 0.02 Ab	0.1 ± 0.01 Ab	0.1 ± 0.02 Ab
	Soft Tissue	0.3 ± 0.07 Aa	0.4 ± 0.09 Ba	0.2 ± 0.01 Ab	0.1 ± 0.01 Ab
	Cosmetic	0.3 ± 0.07 Aa	0.1 ± 0.04 Ab	0.2 ± 0.01 Ab	0.1 ± 0.01 Ab
	PerioGard	0.3 ± 0.07 Aa	0.2 ± 0.02 Ab	0.1 ± 0.02 Ab	0.1 ± 0.01 Ab
SR Phonares II	Distilled water	0.8 ± 0.07 Aa	0.9 ± 0.01 Ab	0.8 ± 0.02 Aab	0.8 ± 0.04 Aa
	Soft Tissue	0.8 ± 0.07 Aa	0.9 ± 0.01 Ab	0.8 ± 0.07 Aab	0.8 ± 0.06 Aab
	Implants	0.8 ± 0.07 Aa	0.9 ± 0.03 Ab	0.8 ± 0.05 Aab	0.8 ± 0.03 Aab
	PerioGard	0.8 ± 0.07 Aa	0.9 ± 0.07 Ab	0.8 ± 0.02 Aab	0.8 ± 0.01 Aab

*Different uppercase letters indicate statistical difference between mouth rinse under the same timepoint for each material. Different lowercase letters indicate statistical difference between timepoints under the same mouth rinse for each material.

CR values of Ivotion Dent, Ivotion Base, and Empress Direct significantly decreased after rinsing simulation with all solutions over timepoints, with no difference among them for each respective material (T3). The translucency of the SR Phonares II artificial teeth was not affected by either factor, while the CR increased only at T1, with no difference regardless of the mouth rinse.

## Discussion

This study evaluated the effect of mouth rinsing simulation using mouth rinses with different compositions on the surface roughness and optical properties of different polymeric dental materials. Based on data analyses, significant modifications on materials’ properties were associated with mouth rinsing using different solutions. Therefore, the postulated null hypothesis was rejected.

Roughness is a property that can influence different features of dental materials, including light scattering, adhesion of microorganisms, and crack initiation [22, 23]. The characteristics of a material surface can be influenced by different factors, such as manufacturing technique, composition, acid solutions, alcohol in beverages, finishing techniques, and aging process [24–27]. Oral care products also contain components, as abrasives or alcohol in different concentrations, which can affect mechanical and optical properties of dental materials [28, 29]. Commercial mouth rinses did not affect the roughness of a milling PMMA for temporary prostheses after a short rinsing (6 h); however, in long-term analysis, increased roughness was observed [30]. In this study, all mouth rinses increased the materials’ roughness, except PerioGard for the Empress Direct resin composite and Soft Tissue and Implants for the SR Phonares II artificial teeth (Figure 2). The composition of mouth rinses may affect polymer materials by promoting the hydrolysis of monomers and fillers, which can lead to the extrusion of fillers from the resin matrix, increasing the surface roughness [31]. The effect of mouth rinses was similar regardless of their composition for the two PMMA CAD/CAM materials Ivotion Base and Ivotion Dent. In contrast, Soft Tissue promoted higher roughness for the Empress Direct resin composite than PerioGard. The matrix composition of the resin composite, which contains Bis-GMA (Bisphenol A glycidyl methacrylate) and UDMA (Urethane Dimethacrylate) monomers that are susceptible to water sorption, may have promoted different physical changes [32, 33]. Conversely, the Soft Tissue and Implants promoted significantly lower roughness for the SR Phonares II artificial teeth compared to PerioGard. The composition of the Soft Tissue and Implants mouth rinses, which are alcohol-free, may have contributed to minimizing changes in artificial tooth surfaces. An increase in roughness was also reported for artificial teeth after exposition to acid solutions and alcohol [25].

As previously mentioned, the surface characteristics are related to microorganism adhesion, and a threshold of Ra 0.2 μm promotes biofilm retention [34]. All materials tested, except the Empress Direct resin composite, reached the critical Ra value after mouth rinsing, which simulated 6 months of use [17, 18]. Considering this result, both professionals and patients must be aware of the importance for regular appointments and polishing procedures for prostheses and, in long-term, for restorations to prevent inflammatory activities and secondary caries.

The roughness is often related to the optical properties, as defects lead to light scattering and facilitate superficial staining [35, 36]. However, despite the increase in roughness, there was an increase in the TP and a decrease in the CR for Ivotion Dent and Empress Direct, while no change for the artificial teeth and Ivotion Base at timepoints T0 and T3. Thus, the increase in roughness did not promote a detrimental effect on the optical properties of the materials. A systematic review has shown that different in vitro mouth rinse protocols were not able to promote clinically unacceptable color changes in resins composite [37]. Additionally, no structural or chemical element changes in enamel or dental materials after long-term exposure to alcohol-containing, low pH, and peroxide-containing mouth rinses were reported in a previous study [12]. Nevertheless, other factors could also contribute to these changes, which warrants further investigations.

Mouth rinsing was simulated on different polymer-based materials for different timepoints. PMMA materials are frequently used in full-mouth rehabilitations, often associated with implants and resin composite restorations. Regular follow-up visits after the installation of prostheses are necessary for occlusion and adaptation adjustments. This allows evaluation of surface texture and polishing procedures to be carried out according to the timepoints proposed in these studies, which represents 45, 90, and 180 days [17, 18]. The surface stability of dental materials is pivotal for preventing caries, periodontal diseases, and inflammatory activities and maintaining optical properties and factors related to the success of rehabilitative treatment.

The three N&W mouth rinses evaluated in this study have different compositions, which align with a wide range of clinical situations. Specifically, the Cosmetic mouth rinse contains fluorides (Sodium Monofluorophosphate and Sodium Fluoride [226 ppm]), which are known to contribute to the prevention of enamel demineralization and the reduction of dental caries [12, 38, 39, 40]. While similar components, as hyaluronic acid, have shown a positive action on tissue repair and wound healing [41, 42], camellia sinensis leaf extract demonstrated to be effective in reducing dentin erosion-abrasion [43] as well as in preventing periodontal disease [44], and melaleuca alternifolia leaf extract presented antimicrobial properties [45]. In addition to biological benefits, the mouth rinse did not promote a detrimental effect in the materials’ optical properties regardless of the rinse composition and material class. Furthermore, the surface roughness increase can be addressed with regular appointments and polishing procedures, which can be easily performed chairside to reestablish surface quality [46–48].

While mouth rinsing simulation provides relevant insights on material behavior, the main limitations of this study include its *in vitro* design that lacked complete simulation of the oral scenario, such as pH fluctuations and temperature variations. Furthermore, the color change was not evaluated. These factors may have a significant impact on dental materials’ behavior, can provide relevant findings, and should be considered for future investigations.

## Conclusion

Based on the findings of this in vitro study, the following conclusions were drawn:

- The surface and optical properties of Ivotion Dent, Ivotion Base, Empress Direct, and SR Phonares II artificial teeth were affected differently by mouth-rinsing simulation.
- Roughness increased after rinsing simulation with all solutions for Ivotion Dent and Ivotion Base.
- The Soft Tissue and Cosmetic mouth rinses increased the roughness of Empress Direct while decreased the Ra for the artificial teeth.
- The TP of Ivotion Dent and Empress Direct increased, while the CR decreased after mouth rinse simulation regardless of the solution.
- Ivotion Base showed higher TP after rinsing with Soft Tissue and Implants compared to the distilled water control.
- TP values of the artificial teeth were not affected by the rinsing simulation and solution.

## Data Availability

The data that support the findings of this study are available from the corresponding author upon reasonable request.

## Key message

Mouth rinses are used for their antimicrobial and anti-inflammatory effects; however, they can affect the optical properties and roughness of dental materials. Given the continuous development of materials and dental care products, it is essential to assess potential changes in their optical and physical properties to ensure their effective use.
